# Crystal structure of 2-chloro-1,3-(2,6-diiso­propyl­phen­yl)-4,5-dihydro-1*H*-imidazol-3-ium tetra­kis­(3,5-tri­fluoro­methyl­phen­yl)borate

**DOI:** 10.1107/S2056989016014584

**Published:** 2016-09-23

**Authors:** Darcie L. Stack, Jason D. Masuda

**Affiliations:** a The Atlantic Centre for Green Chemistry and the Department of Chemistry, Saint Mary’s University, Halifax, Nova Scotia, B3H 3C3, Canada

**Keywords:** crystal structure, *N*-heterocyclic carbene, NHC, 2-chloro imidazolidinium, borate

## Abstract

The title compound, C_27_H_38_ClN_2_^+^·C_32_H_12_BF_24_^−^, was synthesized by reacting the product formed from a previous reaction between 1,3-bis­(2,6-diiso­propyl­phen­yl)imidazolinium-2-carboxyl­ate (SIPrCO_2_), and SOCl_2_, with sodium tetra­kis­[3,5-bis­(tri­fluoro­meth­yl)phen­yl]borate (NaBARF). In the cation, the imidazole ring is in a half-chair conformation and the formerly carbene carbon atom is bonded in a distorted trigonal–planar geometry with N—C—Cl angles of 122.96 (16) and 122.21 (16)° and an N—C—N angle of 114.83 (18)°. In the crystal, weak C—H⋯F hydrogen bonds link the cations and anions, forming a three-dimensional network. In addition, a short Cl⋯F contact of 3.213 Å and several short F⋯F contacts less than the sum of the van der Waals radii [1.47 Å + 1.47 Å = 2.94 Å] are observed. The F atoms of two of the CF_3_ groups were refined as disordered over four sets of sites.

## Abstract 2

The salt compound presented is an example of a 2-chloro imidazolidinium structure where the formerly carbene carbon has a trigonal–planar geometry.

## Chemical context   

The use of main group elements as a way to stabilize singlet carbenes was first investigated in-depth by Bertrand & Reed (1994 ▸), leading to the discovery of the first phosphino silyl carbenes (Igau *et al.*, 1988 ▸) followed by other novel singlet carbenes (Lavallo *et al.*, 2005 ▸; Frey *et al.*, 2007 ▸; Aldeco-Perez *et al.*, 2009 ▸). However, the report of the first ‘bottleable’ crystalline *N*-heterocyclic carbene (NHC) (Arduengo *et al.*, 1991 ▸) initiated a new paradigm in synthetic chemistry (Bourissou *et al.*, 2000 ▸). In particular, NHCs are favoured due to their stability and ease of synthesis. The ability of these stable carbenes to activate small mol­ecules and to help stabilize highly reactive inter­mediates makes this an increasingly desirable area of research. The crystal structure of the compound under investigation incorporates a popular five-membered saturated NHC (known as SIPr) coordinated with a Cl atom attached at the formally carbene atom as a borate salt.
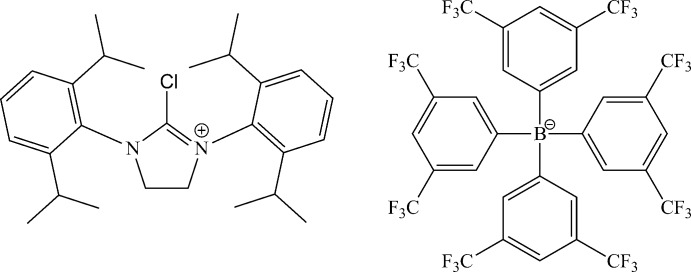


## Structural commentary   

**Figure 1:**
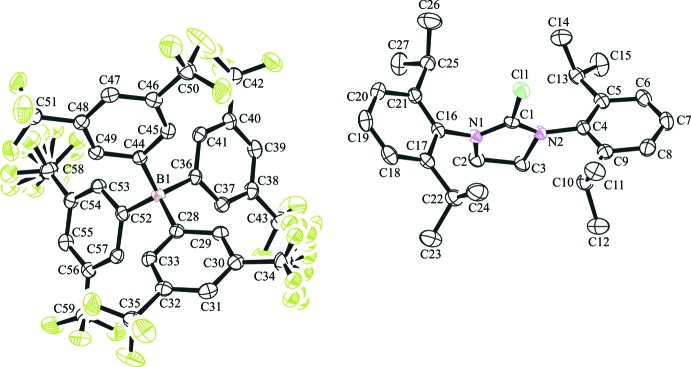
The mol­ecular structure of the title compound showing the atom labelling. Fluorine atom labels and hydrogen atoms have been omitted for clarity. Displacement ellipsoids are drawn at the 50% probability level.

The mol­ecular structure of the title salt compound is shown in Fig. 1 ▸. The formerly carbene carbon has a distorted trigonal–planar geometry and is flanked by the two sterically bulky *N*-diiso­propyl­phenyl groups of the heterocycle. The imidazolidinium ring is in a half-chair conformation having approximate *C*_2_ symmetry. The dihedral angle between the mean planes of the benzene rings is 36.7 (1)°. The isopropyl groups containing C12 and C27 are essentially bis­ected by the plane of the benzene ring to which they are attached, subtending dihedral angles of 116.0 (2)° (C16/C21/C25/C27) and 112.4 (2)° (C4/C9/C10/C12), relative to the *ipso* carbon atoms C4 and C16 while the isopropyl groups containing C15 and C23 deviate significantly from this bis­ected geometry with dihedral angles of 26.1 (2)° (C4/C5/C13/C15) and 46.7 (2)° (C16/C17/C22/C23) relative to the *ipso* carbon atoms C4 and C16. The C1—Cl1 bond length of 1.681 (2) Å is slightly less than the average value of 1.73 Å for a C*sp* ^2^⋯Cl bond length.

## Supra­molecular features   

**Figure 2:**
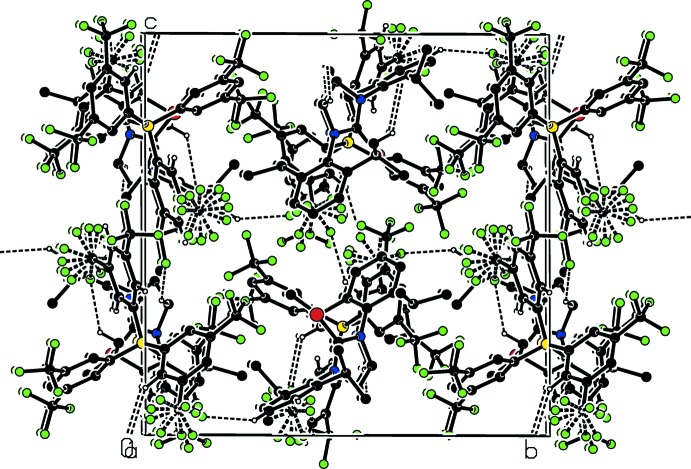
Part of the crystal structure with weak C—H⋯F hydrogen bonds shown as dashed lines.

In the crystal, short-contact H⋯F inter­actions between the isopropyl groups of the NHC and the tri­fluoro­methyl groups of the anion are observed. These are due to weak C—H⋯F hydrogen bonds (Table 1 ▸), which link the cations and anions, forming a three-dimensional network (Fig. 2 ▸). There is one short Cl1⋯F20(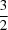 − *x*, −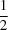 + *y*, 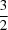 − *z*) contact with a distance of 3.213 (2) Å as well as multiple short F⋯F contacts with lengths less than 2.94 Å.

## Database survey   

A search of the Cambridge Structural Database (CSD; Groom *et al.*, 2016 ▸) revealed two hits for structures which are imidazolidinium salts with *N*-methyl groups in place of the *N*-diiso­propyl­phenyl groups of the title compound. One of the structures contains a tetra­chloro­nickel counter-anion and the other is that of a chloride [XAMQAE (Kremzow *et al.*, 2005 ▸) and SISVUN (Böttcher *et al.*, 2014 ▸)]. The CSD also contains two structures of unsaturated five-membered NHC compounds that contain C—Cl bonds in the C2 position [NUXPOL (Arduengo *et al.*, 1997 ▸) and XOMMER (Kuhn *et al.*, 2002 ▸)].

## Synthesis and crystallization   

In a glovebox, prior to the synthesis of the title compound, SIPrCO_2_ (Zhou *et al.*, 2008 ▸) was reacted with SOCl_2_ in an attempt to synthesize SIPrCOCl_2_. The exact composition of the product was unconfirmed; however, the decision was made to take a portion of this product and move forward to test its chemistry. This product is the primary reagent for the synthesis of the title salt. In a vial equipped with a magnetic stirring bar was placed the resulting product from the SIPrCO_2_/SOCl_2_ reaction (0.0478 g, 9.745 × 10^−2^ mmol), NaBARF (0.0863 g, 9.738 × 10^−2^ mmol) and 5 mL of di­chloro­methane. The mixture was left to stir overnight (18 h) after which the insol­uble solids were removed by filtering the solution into a pre-weighed vial. This was done using a glass pipette containing a small layer of diatomaceous earth. Volatiles were removed *in vacuo*, leaving behind a pale-yellow-coloured solid (0.0596 g, 4.623 × 10^−2^ mmol). The purity of the sample was confirmed using ^1^H NMR spectroscopy in deuterated chloro­form (CDCl_3_). The recrystallization was carried out by evaporation of CDCl_3_, followed by cooling in the freezer overnight, to afford colourless needle-shaped crystals. ^1^H NMR (300 MHz, 298 K, C_6_D_6_): δ 1.26 (d, CH(C*H*_3_)_2_, 12H), 1.33 (d, CH(C*H*_3_)_2_, 12H), 3.84 (sept., C*H*(CH_3_)_2_, 4H), 4.52 (s, C*H*_2_, 4H), 7.34 (d, *m*-Ar-*H*, 4H), 7.50 (s, *p*-Ar-*H*, 4H), 7.56 (t, *p*-Ar-*H*, 2H), 7.68 ppm (t, *m*-Ar-*H*, 8H). ^19^F NMR (282.5 MHz, 298 K, C_6_D_6_): δ −63.1 ppm (s). ^11^B NMR (96.3 MHz, 298 K, C_6_D_6_): δ −6.18 ppm (s). Tri­fluoro­toluene was used as an external reference for the ^19^F NMR spectrum and boron trifluoride diethyl etherate was used as the external reference for the ^11^B NMR spectrum.

## Refinement   

Crystal data, data collection and structure refinement details are summarized in Table 2 ▸. Hydrogen atoms were included at geometrically idealized positions and were included in a riding-motion approximation. For the methyl groups, the dihedral angle of the idealized tetra­hedral CH_3_ fragment was allowed to refine.

Prior to final refinement, there was significant disorder associated with one of the CF_3_ groups attached to each of C34 and C58. After trying to assess whether the groups had two components of a disorder, it became clear that each of these CF_3_ groups actually had four components of disorder that needed to be resolved. In order to do this, the SUMP command was applied to all of the fluorine atoms involved. This involved grouping the four components into PART 1, PART 2, PART 3, and PART 4, respectively, and assigning a free variable to each of the individual parts, where the weighted sum of the free variables was set to equal 1.0 (C58: 0.5: 0.3: 0.1: 0.1 and C34: 0.4: 0.3: 0.2: 0.1). Following refinement using the SUMP command, the EADP command was applied, which allowed for all of the anisotropic parameters of the fluorine ellipsoids to be similar in size. Lastly, the SADI command was applied to each of the affected C—F bonds in the disordered CF_3_ groups in order to have similar bond lengths for each of the disordered F atoms (*i.e.* the bond lengths were approximately equal for C58—F16*A*–*D*, C58—F17*A*–*D*, *etc*). The combination of these commands allowed for complete refinement of the CF_3_ disorder.

## Supplementary Material

Crystal structure: contains datablock(s) I. DOI: 10.1107/S2056989016014584/lh5822sup1.cif

Structure factors: contains datablock(s) I. DOI: 10.1107/S2056989016014584/lh5822Isup2.hkl

CCDC reference: 1504219

Additional supporting information: crystallographic information; 3D view; checkCIF report

## Figures and Tables

**Table 1:** Hydrogen-bond geometry (Å, °)

*D*—H⋯*A*	*D*—H	H⋯*A*	*D*⋯*A*	*D*—H⋯*A*
C3—H3*A*⋯F15^i^	0.99	2.43	3.256 (3)	141
C12—H12*A*⋯F17*C* ^ii^	0.98	2.53	3.269 (12)	132
C19—H19⋯F8*D* ^iii^	0.95	2.48	3.297 (18)	144
C29—H29⋯F9*D* ^iii^	0.95	2.35	3.180 (14)	146

Symmetry codes: (i) 
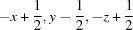
; (ii) 
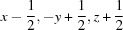
; (iii) 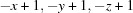.

**Table 2:** Experimental details

Crystal data	
Chemical formula	C_17_H_38_ClN_2_^+^·C_32_H_12_BF_24_
*M*_r_	1289.27
Crystal system, space group	Monoclinic, *P*2_1_/*n*
Temperature (K)	125
*a*, *b*, *c* (Å)	18.5025 (12), 17.8739 (12), 19.7857 (13)
β (°)	116.428 (1)
*V* (Å^3^)	5859.5 (7)
*Z*	4
Radiation type	Mo *K*α
μ (mm^−1^)	0.18
Crystal size (mm)	0.39 × 0.38 × 0.08

Data collection	
Diffractometer	Siemens/Bruker APEXII
Absorption correction	Multi-scan (*SADABS*; Bruker, 2008 ▸)
*T*_min_, *T*_max_	0.660, 0.746
No. of measured, independent and observed [*I* > 2σ(*I*)] reflections	40063, 10921, 8363
*R*_int_	0.033
(sin θ/λ)_max_ (Å^−1^)	0.606

Refinement	
*R*[*F* ^2^ > 2σ(*F* ^2^)], *wR*(*F* ^2^), *S*	0.044, 0.113, 1.02
No. of reflections	10921
No. of parameters	830
No. of restraints	38
H-atom treatment	H-atom parameters constrained
Δρ_max_, Δρ_min_ (e Å^−3^)	0.52, −0.45

Computer programs: *APEX2* and *SAINT* (Bruker, 2008 ▸), *XS* in *SHELXTL* (Sheldrick, 2008 ▸), *SHELXL2014/7* (Sheldrick, 2015 ▸), *ORTEP-3 for Windows* (Farrugia, 2012 ▸), *PLATON* (Spek, 2009 ▸) and *publCIF* (Westrip, 2010 ▸).

## supplementary crystallographic information

### Crystal data

**Table:**

C_17_H_38_ClN_2_^+^·C_32_H_12_BF_24_	*F*(000) = 2624
*M**_r_* = 1289.27	*D*_x_ = 1.461 Mg m^−^^3^
Monoclinic, *P*2_1_/*n*	Mo *K*α radiation, λ = 0.71073 Å
*a* = 18.5025 (12) Å	Cell parameters from 9997 reflections
*b* = 17.8739 (12) Å	θ = 2.3–27.8°
*c* = 19.7857 (13) Å	µ = 0.18 mm^−^^1^
β = 116.428 (1)°	*T* = 125 K
*V* = 5859.5 (7) Å^3^	Needle, colourless
*Z* = 4	0.39 × 0.38 × 0.08 mm

### Data collection

**Table:**

Siemens/Bruker APEXII diffractometer	8363 reflections with *I* > 2σ(*I*)
φ and ω scans	*R*_int_ = 0.033
Absorption correction: multi-scan (SADABS; Bruker, 2008)	θ_max_ = 25.5°, θ_min_ = 2.0°
*T*_min_ = 0.660, *T*_max_ = 0.746	*h* = −22→22
40063 measured reflections	*k* = −21→21
10921 independent reflections	*l* = −23→23

### Refinement

**Table:**

Refinement on *F*^2^	38 restraints
Least-squares matrix: full	Hydrogen site location: inferred from neighbouring sites
*R*[*F*^2^ > 2σ(*F*^2^)] = 0.044	H-atom parameters constrained
*wR*(*F*^2^) = 0.113	*w* = 1/[σ^2^(*F*_o_^2^) + (0.0464*P*)^2^ + 4.6564*P*] where *P* = (*F*_o_^2^ + 2*F*_c_^2^)/3
*S* = 1.02	(Δ/σ)_max_ < 0.001
10921 reflections	Δρ_max_ = 0.52 e Å^−^^3^
830 parameters	Δρ_min_ = −0.45 e Å^−^^3^

### Special details

**Table:**

Geometry. All esds (except the esd in the dihedral angle between two l.s. planes) are estimated using the full covariance matrix. The cell esds are taken into account individually in the estimation of esds in distances, angles and torsion angles; correlations between esds in cell parameters are only used when they are defined by crystal symmetry. An approximate (isotropic) treatment of cell esds is used for estimating esds involving l.s. planes.

### Fractional atomic coordinates and isotropic or equivalent isotropic displacement parameters (Å^2^)

**Table:**

	*x*	*y*	*z*	*U*_iso_*/*U*_eq_	Occ. (<1)
Cl1	0.57779 (3)	0.07669 (3)	0.80176 (3)	0.03051 (14)	
F1	0.71125 (9)	0.20864 (8)	0.39608 (8)	0.0499 (4)	
F2	0.76498 (9)	0.31577 (9)	0.43499 (10)	0.0592 (5)	
F3	0.71913 (9)	0.25293 (9)	0.49856 (8)	0.0469 (4)	
F4	0.38941 (10)	0.28263 (11)	0.39231 (9)	0.0641 (5)	
F5	0.41659 (10)	0.18771 (9)	0.34260 (12)	0.0690 (5)	
F6	0.34175 (9)	0.27432 (9)	0.27269 (8)	0.0507 (4)	
F10	0.33308 (9)	0.78402 (7)	0.23647 (8)	0.0459 (4)	
F11	0.26822 (9)	0.70653 (8)	0.14865 (9)	0.0537 (4)	
F12	0.38410 (10)	0.74577 (8)	0.16469 (9)	0.0497 (4)	
F13	0.25354 (8)	0.41455 (8)	0.01425 (8)	0.0445 (4)	
F14	0.27430 (8)	0.52975 (8)	0.00069 (8)	0.0385 (3)	
F15	0.28655 (8)	0.44738 (9)	−0.07256 (7)	0.0470 (4)	
F19	0.82744 (9)	0.65439 (9)	0.51987 (8)	0.0488 (4)	
F20	0.81541 (11)	0.53617 (9)	0.52327 (8)	0.0657 (5)	
F21	0.72766 (10)	0.60866 (10)	0.53192 (8)	0.0561 (5)	
F22	0.67492 (8)	0.67563 (8)	0.16727 (7)	0.0426 (4)	
F23	0.75829 (10)	0.73793 (8)	0.26143 (9)	0.0490 (4)	
F24	0.79387 (9)	0.63343 (9)	0.23298 (9)	0.0492 (4)	
N1	0.49006 (10)	0.03298 (9)	0.66179 (9)	0.0219 (4)	
N2	0.47084 (10)	−0.03195 (9)	0.74765 (9)	0.0225 (4)	
C1	0.50909 (12)	0.02306 (11)	0.73361 (11)	0.0219 (4)	
C2	0.43398 (13)	−0.02710 (12)	0.61751 (12)	0.0273 (5)	
H2A	0.4616	−0.0647	0.6004	0.033*	
H2B	0.3871	−0.0066	0.5731	0.033*	
C3	0.40817 (13)	−0.06074 (13)	0.67455 (11)	0.0275 (5)	
H3A	0.3537	−0.0433	0.6651	0.033*	
H3B	0.4085	−0.1161	0.6731	0.033*	
C4	0.46845 (12)	−0.04819 (12)	0.81830 (11)	0.0239 (5)	
C5	0.50674 (13)	−0.11362 (12)	0.85634 (12)	0.0262 (5)	
C6	0.49935 (14)	−0.13078 (13)	0.92158 (12)	0.0308 (5)	
H6	0.5246	−0.1745	0.9493	0.037*	
C7	0.45600 (14)	−0.08535 (13)	0.94660 (12)	0.0317 (5)	
H7	0.4515	−0.0984	0.9911	0.038*	
C8	0.41908 (14)	−0.02121 (13)	0.90777 (12)	0.0311 (5)	
H8	0.3898	0.0095	0.9261	0.037*	
C9	0.42415 (13)	−0.00086 (12)	0.84220 (12)	0.0261 (5)	
C10	0.38168 (13)	0.06998 (13)	0.80057 (13)	0.0304 (5)	
H10	0.3933	0.0762	0.7561	0.037*	
C11	0.41371 (15)	0.13920 (13)	0.85073 (14)	0.0378 (6)	
H11A	0.3886	0.1842	0.8213	0.057*	
H11B	0.4724	0.1421	0.8695	0.057*	
H11C	0.4006	0.1356	0.8935	0.057*	
C12	0.29009 (14)	0.06342 (15)	0.77105 (15)	0.0431 (6)	
H12A	0.2643	0.1097	0.7450	0.065*	
H12B	0.2773	0.0550	0.8135	0.065*	
H12C	0.2701	0.0213	0.7358	0.065*	
C13	0.55597 (14)	−0.16215 (13)	0.82903 (13)	0.0312 (5)	
H13	0.5292	−0.1605	0.7726	0.037*	
C14	0.64129 (15)	−0.13026 (15)	0.85628 (15)	0.0419 (6)	
H14A	0.6718	−0.1614	0.8372	0.063*	
H14B	0.6686	−0.1300	0.9116	0.063*	
H14C	0.6380	−0.0790	0.8375	0.063*	
C15	0.56033 (19)	−0.24404 (14)	0.85261 (16)	0.0466 (7)	
H15A	0.5056	−0.2642	0.8343	0.070*	
H15B	0.5883	−0.2477	0.9078	0.070*	
H15C	0.5900	−0.2728	0.8309	0.070*	
C16	0.52057 (13)	0.08945 (11)	0.62913 (11)	0.0228 (4)	
C17	0.47416 (13)	0.15411 (12)	0.60097 (11)	0.0252 (5)	
C18	0.50294 (15)	0.20684 (12)	0.56719 (12)	0.0313 (5)	
H18	0.4733	0.2516	0.5476	0.038*	
C19	0.57381 (15)	0.19514 (13)	0.56161 (13)	0.0335 (5)	
H19	0.5922	0.2317	0.5380	0.040*	
C20	0.61821 (14)	0.13085 (13)	0.59001 (12)	0.0307 (5)	
H20	0.6669	0.1238	0.5858	0.037*	
C21	0.59283 (13)	0.07586 (12)	0.62487 (11)	0.0253 (5)	
C22	0.39771 (14)	0.16936 (13)	0.60966 (13)	0.0310 (5)	
H22	0.3813	0.1213	0.6247	0.037*	
C23	0.32750 (15)	0.19532 (16)	0.53670 (14)	0.0439 (6)	
H23A	0.3406	0.2439	0.5219	0.066*	
H23B	0.2790	0.2002	0.5445	0.066*	
H23C	0.3178	0.1586	0.4968	0.066*	
C24	0.41498 (16)	0.22592 (16)	0.67338 (14)	0.0432 (6)	
H24A	0.4300	0.2742	0.6597	0.065*	
H24B	0.4594	0.2075	0.7200	0.065*	
H24C	0.3666	0.2322	0.6811	0.065*	
C25	0.64282 (14)	0.00569 (13)	0.65617 (13)	0.0318 (5)	
H25	0.6175	−0.0237	0.6830	0.038*	
C26	0.72921 (16)	0.02464 (17)	0.71340 (17)	0.0543 (8)	
H26A	0.7561	0.0522	0.6881	0.081*	
H26B	0.7589	−0.0217	0.7349	0.081*	
H26C	0.7280	0.0556	0.7538	0.081*	
C27	0.64207 (18)	−0.04305 (14)	0.59237 (15)	0.0442 (7)	
H27A	0.5862	−0.0555	0.5573	0.066*	
H27B	0.6724	−0.0892	0.6135	0.066*	
H27C	0.6671	−0.0157	0.5653	0.066*	
C28	0.46452 (12)	0.54706 (11)	0.28500 (11)	0.0214 (4)	
C29	0.44991 (12)	0.54002 (12)	0.34834 (11)	0.0236 (4)	
H29	0.4736	0.4994	0.3819	0.028*	
C30	0.40201 (13)	0.59036 (12)	0.36383 (12)	0.0251 (5)	
C31	0.36629 (13)	0.65038 (12)	0.31637 (12)	0.0271 (5)	
H31	0.3326	0.6843	0.3263	0.033*	
C32	0.38103 (12)	0.65968 (12)	0.25381 (12)	0.0240 (5)	
C33	0.42937 (12)	0.60951 (11)	0.23918 (12)	0.0228 (4)	
H33	0.4391	0.6177	0.1965	0.027*	
C34	0.39268 (15)	0.58098 (14)	0.43418 (14)	0.0357 (6)	
C35	0.34218 (14)	0.72339 (13)	0.20124 (13)	0.0305 (5)	
C36	0.53982 (12)	0.41235 (11)	0.31391 (11)	0.0215 (4)	
C37	0.47298 (13)	0.37166 (12)	0.30880 (11)	0.0248 (5)	
H37	0.4207	0.3928	0.2823	0.030*	
C38	0.48060 (13)	0.30127 (12)	0.34134 (12)	0.0274 (5)	
C39	0.55582 (13)	0.26877 (12)	0.38019 (12)	0.0270 (5)	
H39	0.5612	0.2208	0.4026	0.032*	
C40	0.62297 (13)	0.30745 (12)	0.38578 (11)	0.0243 (5)	
C41	0.61488 (13)	0.37765 (12)	0.35287 (11)	0.0230 (4)	
H41	0.6619	0.4028	0.3570	0.028*	
C42	0.70419 (14)	0.27203 (12)	0.42822 (13)	0.0299 (5)	
C43	0.40729 (15)	0.26223 (14)	0.33705 (14)	0.0367 (6)	
C44	0.61133 (12)	0.54189 (11)	0.30350 (11)	0.0207 (4)	
C45	0.65695 (13)	0.55098 (12)	0.38128 (12)	0.0235 (4)	
H45	0.6407	0.5253	0.4143	0.028*	
C46	0.72526 (13)	0.59620 (12)	0.41220 (12)	0.0249 (5)	
C47	0.75007 (13)	0.63552 (12)	0.36600 (12)	0.0252 (5)	
H47	0.7966	0.6665	0.3867	0.030*	
C48	0.70505 (12)	0.62841 (11)	0.28859 (12)	0.0226 (4)	
C49	0.63740 (12)	0.58233 (11)	0.25804 (11)	0.0220 (4)	
H49	0.6081	0.5783	0.2047	0.026*	
C50	0.77279 (15)	0.59941 (14)	0.49580 (13)	0.0351 (6)	
C51	0.73266 (13)	0.66856 (12)	0.23798 (12)	0.0267 (5)	
C52	0.49542 (12)	0.46987 (11)	0.18099 (11)	0.0215 (4)	
C53	0.54765 (12)	0.43508 (12)	0.15634 (12)	0.0235 (4)	
H53	0.6028	0.4289	0.1912	0.028*	
C54	0.52144 (13)	0.40933 (12)	0.08269 (12)	0.0248 (5)	
C55	0.44147 (13)	0.41754 (12)	0.03016 (12)	0.0259 (5)	
H55	0.4233	0.4004	−0.0202	0.031*	
C56	0.38899 (13)	0.45129 (11)	0.05310 (11)	0.0233 (4)	
C57	0.41534 (12)	0.47617 (11)	0.12721 (11)	0.0224 (4)	
H57	0.3774	0.4981	0.1414	0.027*	
C59	0.30216 (14)	0.46077 (13)	−0.00085 (12)	0.0305 (5)	
B1	0.52749 (14)	0.49284 (13)	0.27032 (13)	0.0212 (5)	
C58	0.57881 (14)	0.36974 (13)	0.06062 (12)	0.0334 (5)	
F16A	0.5578 (3)	0.3839 (2)	−0.01354 (12)	0.0362 (4)	0.457 (3)
F17A	0.5790 (3)	0.29508 (11)	0.0682 (3)	0.0362 (4)	0.457 (3)
F18A	0.65707 (14)	0.3914 (3)	0.0972 (3)	0.0362 (4)	0.457 (3)
F16B	0.5558 (5)	0.3618 (4)	−0.01471 (13)	0.0362 (4)	0.315 (3)
F17B	0.5990 (4)	0.30242 (19)	0.0940 (3)	0.0362 (4)	0.315 (3)
F18B	0.6470 (2)	0.4117 (3)	0.0875 (4)	0.0362 (4)	0.315 (3)
F16C	0.5496 (8)	0.2997 (4)	0.0363 (9)	0.0362 (4)	0.118 (3)
F17C	0.6562 (3)	0.3652 (9)	0.1125 (6)	0.0362 (4)	0.118 (3)
F18C	0.5837 (9)	0.3942 (8)	−0.0020 (5)	0.0362 (4)	0.118 (3)
F16D	0.5391 (7)	0.3310 (8)	−0.0051 (6)	0.0362 (4)	0.110 (3)
F17D	0.6205 (9)	0.3188 (6)	0.1136 (6)	0.0362 (4)	0.110 (3)
F18D	0.6404 (6)	0.4108 (7)	0.0606 (9)	0.0362 (4)	0.110 (3)
F7A	0.3939 (5)	0.51089 (14)	0.4606 (3)	0.0351 (5)	0.398 (3)
F8A	0.3370 (4)	0.6223 (4)	0.4436 (4)	0.0351 (5)	0.398 (3)
F9A	0.4609 (2)	0.6116 (3)	0.49079 (19)	0.0351 (5)	0.398 (3)
F7B	0.3620 (4)	0.51232 (18)	0.4344 (3)	0.0351 (5)	0.338 (3)
F8B	0.3334 (5)	0.6281 (5)	0.4284 (4)	0.0351 (5)	0.338 (3)
F9B	0.4625 (2)	0.5889 (3)	0.4999 (2)	0.0351 (5)	0.338 (3)
F7C	0.3999 (9)	0.50701 (19)	0.4506 (7)	0.0351 (5)	0.190 (3)
F8C	0.3188 (3)	0.5964 (6)	0.4282 (5)	0.0351 (5)	0.190 (3)
F9C	0.4455 (4)	0.6156 (6)	0.4995 (4)	0.0351 (5)	0.190 (3)
F7D	0.3279 (8)	0.5350 (9)	0.4112 (10)	0.0351 (5)	0.0734 (19)
F8D	0.3577 (12)	0.6383 (9)	0.4527 (14)	0.0351 (5)	0.0734 (19)
F9D	0.4442 (10)	0.5377 (10)	0.4934 (7)	0.0351 (5)	0.0734 (19)

### Atomic displacement parameters (Å^2^)

**Table:**

	*U*^11^	*U*^22^	*U*^33^	*U*^12^	*U*^13^	*U*^23^
Cl1	0.0307 (3)	0.0299 (3)	0.0250 (3)	−0.0082 (2)	0.0070 (2)	−0.0015 (2)
F1	0.0515 (9)	0.0446 (9)	0.0496 (9)	0.0195 (7)	0.0189 (8)	−0.0124 (7)
F2	0.0289 (8)	0.0459 (9)	0.0903 (13)	0.0045 (7)	0.0153 (8)	0.0269 (9)
F3	0.0488 (9)	0.0569 (10)	0.0318 (8)	0.0221 (7)	0.0149 (7)	0.0079 (7)
F4	0.0546 (10)	0.1007 (14)	0.0528 (10)	−0.0322 (10)	0.0381 (9)	−0.0135 (9)
F5	0.0544 (10)	0.0349 (9)	0.1155 (16)	−0.0146 (8)	0.0357 (11)	0.0160 (9)
F6	0.0337 (8)	0.0657 (11)	0.0449 (9)	−0.0190 (7)	0.0105 (7)	0.0062 (8)
F10	0.0632 (10)	0.0234 (7)	0.0480 (9)	0.0094 (7)	0.0220 (8)	0.0000 (6)
F11	0.0391 (9)	0.0390 (9)	0.0491 (9)	0.0009 (7)	−0.0108 (7)	0.0055 (7)
F12	0.0595 (10)	0.0430 (9)	0.0576 (10)	0.0171 (7)	0.0360 (8)	0.0254 (7)
F13	0.0288 (7)	0.0461 (9)	0.0550 (9)	−0.0129 (6)	0.0153 (7)	−0.0047 (7)
F14	0.0290 (7)	0.0378 (8)	0.0402 (8)	0.0058 (6)	0.0077 (6)	−0.0012 (6)
F15	0.0330 (8)	0.0726 (11)	0.0245 (7)	0.0039 (7)	0.0029 (6)	−0.0126 (7)
F19	0.0449 (9)	0.0591 (10)	0.0325 (8)	−0.0212 (8)	0.0082 (7)	−0.0156 (7)
F20	0.0818 (12)	0.0504 (10)	0.0300 (8)	0.0155 (9)	−0.0065 (8)	0.0039 (7)
F21	0.0561 (10)	0.0880 (13)	0.0281 (7)	−0.0215 (9)	0.0221 (7)	−0.0141 (8)
F22	0.0393 (8)	0.0531 (9)	0.0333 (7)	−0.0058 (7)	0.0143 (6)	0.0144 (7)
F23	0.0696 (11)	0.0312 (8)	0.0549 (9)	−0.0185 (7)	0.0354 (8)	−0.0028 (7)
F24	0.0502 (9)	0.0524 (9)	0.0659 (10)	0.0230 (8)	0.0448 (8)	0.0260 (8)
N1	0.0216 (9)	0.0207 (9)	0.0201 (9)	−0.0012 (7)	0.0064 (7)	0.0005 (7)
N2	0.0224 (9)	0.0213 (9)	0.0202 (9)	−0.0041 (7)	0.0061 (7)	0.0003 (7)
C1	0.0210 (10)	0.0185 (10)	0.0231 (11)	0.0029 (8)	0.0070 (9)	0.0003 (8)
C2	0.0288 (12)	0.0262 (12)	0.0228 (11)	−0.0052 (9)	0.0078 (9)	−0.0034 (9)
C3	0.0264 (11)	0.0291 (12)	0.0218 (11)	−0.0070 (9)	0.0061 (9)	−0.0016 (9)
C4	0.0224 (11)	0.0266 (11)	0.0196 (10)	−0.0074 (9)	0.0068 (9)	0.0004 (8)
C5	0.0264 (11)	0.0242 (11)	0.0241 (11)	−0.0069 (9)	0.0077 (9)	−0.0014 (9)
C6	0.0353 (13)	0.0272 (12)	0.0253 (11)	−0.0045 (10)	0.0093 (10)	0.0037 (9)
C7	0.0353 (13)	0.0363 (13)	0.0229 (11)	−0.0113 (10)	0.0126 (10)	0.0006 (10)
C8	0.0296 (12)	0.0358 (13)	0.0286 (12)	−0.0073 (10)	0.0137 (10)	−0.0054 (10)
C9	0.0225 (11)	0.0273 (12)	0.0251 (11)	−0.0069 (9)	0.0074 (9)	−0.0021 (9)
C10	0.0290 (12)	0.0307 (12)	0.0305 (12)	0.0018 (10)	0.0123 (10)	0.0011 (10)
C11	0.0399 (14)	0.0297 (13)	0.0384 (14)	0.0023 (11)	0.0124 (11)	−0.0016 (10)
C12	0.0302 (13)	0.0440 (15)	0.0480 (15)	0.0028 (11)	0.0110 (12)	0.0026 (12)
C13	0.0363 (13)	0.0264 (12)	0.0274 (12)	0.0007 (10)	0.0112 (10)	0.0032 (9)
C14	0.0324 (13)	0.0399 (15)	0.0492 (15)	0.0057 (11)	0.0142 (12)	0.0022 (12)
C15	0.0691 (19)	0.0267 (13)	0.0488 (16)	0.0030 (13)	0.0305 (15)	0.0038 (11)
C16	0.0262 (11)	0.0220 (11)	0.0179 (10)	−0.0025 (9)	0.0077 (9)	0.0009 (8)
C17	0.0287 (12)	0.0231 (11)	0.0194 (10)	0.0014 (9)	0.0067 (9)	−0.0009 (8)
C18	0.0439 (14)	0.0206 (11)	0.0266 (11)	0.0033 (10)	0.0131 (11)	0.0033 (9)
C19	0.0500 (15)	0.0257 (12)	0.0292 (12)	−0.0057 (11)	0.0215 (11)	0.0017 (9)
C20	0.0336 (13)	0.0325 (13)	0.0312 (12)	−0.0034 (10)	0.0191 (10)	−0.0011 (10)
C21	0.0276 (11)	0.0271 (12)	0.0210 (10)	0.0009 (9)	0.0107 (9)	0.0007 (9)
C22	0.0326 (12)	0.0246 (12)	0.0345 (12)	0.0067 (10)	0.0139 (10)	0.0032 (9)
C23	0.0353 (14)	0.0463 (16)	0.0400 (14)	0.0102 (12)	0.0077 (12)	0.0013 (12)
C24	0.0382 (14)	0.0502 (16)	0.0384 (14)	0.0125 (12)	0.0145 (12)	−0.0052 (12)
C25	0.0328 (12)	0.0318 (13)	0.0349 (12)	0.0071 (10)	0.0187 (11)	0.0084 (10)
C26	0.0375 (15)	0.0547 (18)	0.0590 (18)	0.0144 (13)	0.0109 (14)	0.0113 (15)
C27	0.0641 (18)	0.0307 (14)	0.0510 (16)	0.0119 (13)	0.0375 (15)	0.0066 (12)
C28	0.0192 (10)	0.0233 (11)	0.0194 (10)	−0.0035 (8)	0.0066 (8)	−0.0028 (8)
C29	0.0214 (11)	0.0242 (11)	0.0236 (11)	−0.0007 (9)	0.0085 (9)	−0.0014 (9)
C30	0.0232 (11)	0.0271 (12)	0.0244 (11)	−0.0016 (9)	0.0101 (9)	−0.0039 (9)
C31	0.0233 (11)	0.0257 (12)	0.0318 (12)	−0.0004 (9)	0.0119 (10)	−0.0075 (9)
C32	0.0207 (11)	0.0214 (11)	0.0256 (11)	−0.0030 (8)	0.0065 (9)	−0.0024 (8)
C33	0.0219 (10)	0.0232 (11)	0.0231 (10)	−0.0048 (8)	0.0098 (9)	−0.0018 (8)
C34	0.0395 (14)	0.0370 (14)	0.0373 (13)	0.0063 (11)	0.0231 (12)	0.0004 (11)
C35	0.0292 (12)	0.0267 (12)	0.0317 (12)	0.0024 (9)	0.0100 (10)	−0.0010 (10)
C36	0.0253 (11)	0.0221 (11)	0.0191 (10)	−0.0019 (8)	0.0118 (9)	−0.0028 (8)
C37	0.0247 (11)	0.0271 (12)	0.0226 (10)	−0.0011 (9)	0.0105 (9)	−0.0012 (9)
C38	0.0309 (12)	0.0259 (12)	0.0271 (11)	−0.0072 (9)	0.0145 (10)	−0.0025 (9)
C39	0.0373 (13)	0.0209 (11)	0.0251 (11)	−0.0007 (9)	0.0159 (10)	−0.0001 (9)
C40	0.0304 (12)	0.0223 (11)	0.0219 (10)	0.0002 (9)	0.0132 (9)	−0.0023 (8)
C41	0.0247 (11)	0.0231 (11)	0.0242 (10)	−0.0019 (9)	0.0137 (9)	−0.0029 (8)
C42	0.0341 (13)	0.0235 (12)	0.0338 (13)	0.0042 (10)	0.0167 (11)	0.0006 (9)
C43	0.0362 (14)	0.0363 (14)	0.0369 (13)	−0.0082 (11)	0.0157 (12)	0.0017 (11)
C44	0.0225 (10)	0.0192 (10)	0.0222 (10)	0.0037 (8)	0.0115 (9)	0.0001 (8)
C45	0.0263 (11)	0.0214 (11)	0.0256 (11)	0.0013 (9)	0.0140 (9)	0.0008 (8)
C46	0.0249 (11)	0.0233 (11)	0.0244 (11)	0.0026 (9)	0.0092 (9)	−0.0028 (9)
C47	0.0216 (11)	0.0216 (11)	0.0309 (12)	−0.0003 (9)	0.0103 (9)	−0.0027 (9)
C48	0.0229 (11)	0.0177 (10)	0.0290 (11)	0.0035 (8)	0.0131 (9)	0.0009 (8)
C49	0.0224 (10)	0.0220 (11)	0.0213 (10)	0.0038 (8)	0.0096 (9)	−0.0014 (8)
C50	0.0372 (13)	0.0352 (13)	0.0271 (12)	−0.0042 (11)	0.0089 (11)	−0.0037 (10)
C51	0.0259 (12)	0.0230 (11)	0.0334 (12)	0.0007 (9)	0.0152 (10)	0.0015 (9)
C52	0.0248 (11)	0.0172 (10)	0.0228 (10)	−0.0021 (8)	0.0109 (9)	0.0018 (8)
C53	0.0209 (10)	0.0244 (11)	0.0239 (10)	−0.0009 (8)	0.0088 (9)	0.0005 (8)
C54	0.0280 (11)	0.0222 (11)	0.0270 (11)	−0.0015 (9)	0.0148 (9)	−0.0002 (9)
C55	0.0310 (12)	0.0240 (11)	0.0232 (11)	−0.0033 (9)	0.0126 (10)	−0.0038 (9)
C56	0.0250 (11)	0.0200 (11)	0.0244 (11)	−0.0027 (8)	0.0104 (9)	−0.0004 (8)
C57	0.0231 (11)	0.0204 (11)	0.0255 (11)	−0.0019 (8)	0.0125 (9)	0.0007 (8)
C59	0.0274 (12)	0.0338 (13)	0.0284 (12)	−0.0036 (10)	0.0107 (10)	−0.0062 (10)
B1	0.0208 (12)	0.0223 (12)	0.0199 (11)	0.0010 (9)	0.0085 (10)	0.0010 (9)
C58	0.0317 (13)	0.0377 (14)	0.0305 (12)	0.0009 (10)	0.0136 (10)	−0.0048 (10)
F16A	0.0396 (8)	0.0351 (10)	0.0406 (8)	0.0062 (7)	0.0239 (7)	−0.0056 (7)
F17A	0.0396 (8)	0.0351 (10)	0.0406 (8)	0.0062 (7)	0.0239 (7)	−0.0056 (7)
F18A	0.0396 (8)	0.0351 (10)	0.0406 (8)	0.0062 (7)	0.0239 (7)	−0.0056 (7)
F16B	0.0396 (8)	0.0351 (10)	0.0406 (8)	0.0062 (7)	0.0239 (7)	−0.0056 (7)
F17B	0.0396 (8)	0.0351 (10)	0.0406 (8)	0.0062 (7)	0.0239 (7)	−0.0056 (7)
F18B	0.0396 (8)	0.0351 (10)	0.0406 (8)	0.0062 (7)	0.0239 (7)	−0.0056 (7)
F16C	0.0396 (8)	0.0351 (10)	0.0406 (8)	0.0062 (7)	0.0239 (7)	−0.0056 (7)
F17C	0.0396 (8)	0.0351 (10)	0.0406 (8)	0.0062 (7)	0.0239 (7)	−0.0056 (7)
F18C	0.0396 (8)	0.0351 (10)	0.0406 (8)	0.0062 (7)	0.0239 (7)	−0.0056 (7)
F16D	0.0396 (8)	0.0351 (10)	0.0406 (8)	0.0062 (7)	0.0239 (7)	−0.0056 (7)
F17D	0.0396 (8)	0.0351 (10)	0.0406 (8)	0.0062 (7)	0.0239 (7)	−0.0056 (7)
F18D	0.0396 (8)	0.0351 (10)	0.0406 (8)	0.0062 (7)	0.0239 (7)	−0.0056 (7)
F7A	0.0448 (8)	0.0450 (9)	0.0241 (9)	0.0091 (6)	0.0230 (7)	0.0062 (6)
F8A	0.0448 (8)	0.0450 (9)	0.0241 (9)	0.0091 (6)	0.0230 (7)	0.0062 (6)
F9A	0.0448 (8)	0.0450 (9)	0.0241 (9)	0.0091 (6)	0.0230 (7)	0.0062 (6)
F7B	0.0448 (8)	0.0450 (9)	0.0241 (9)	0.0091 (6)	0.0230 (7)	0.0062 (6)
F8B	0.0448 (8)	0.0450 (9)	0.0241 (9)	0.0091 (6)	0.0230 (7)	0.0062 (6)
F9B	0.0448 (8)	0.0450 (9)	0.0241 (9)	0.0091 (6)	0.0230 (7)	0.0062 (6)
F7C	0.0448 (8)	0.0450 (9)	0.0241 (9)	0.0091 (6)	0.0230 (7)	0.0062 (6)
F8C	0.0448 (8)	0.0450 (9)	0.0241 (9)	0.0091 (6)	0.0230 (7)	0.0062 (6)
F9C	0.0448 (8)	0.0450 (9)	0.0241 (9)	0.0091 (6)	0.0230 (7)	0.0062 (6)
F7D	0.0448 (8)	0.0450 (9)	0.0241 (9)	0.0091 (6)	0.0230 (7)	0.0062 (6)
F8D	0.0448 (8)	0.0450 (9)	0.0241 (9)	0.0091 (6)	0.0230 (7)	0.0062 (6)
F9D	0.0448 (8)	0.0450 (9)	0.0241 (9)	0.0091 (6)	0.0230 (7)	0.0062 (6)

### Geometric parameters (Å, º)

**Table:**

Cl1—C1	1.681 (2)	C30—C34	1.486 (3)
F1—C42	1.334 (3)	C31—C32	1.391 (3)
F2—C42	1.327 (3)	C32—C33	1.385 (3)
F3—C42	1.337 (3)	C32—C35	1.493 (3)
F4—C43	1.327 (3)	C34—F8C	1.346 (3)
F5—C43	1.341 (3)	C34—F8A	1.347 (3)
F6—C43	1.328 (3)	C34—F8D	1.347 (3)
F10—C35	1.339 (3)	C34—F8B	1.347 (3)
F11—C35	1.334 (3)	C34—F7B	1.353 (3)
F12—C35	1.336 (3)	C34—F7A	1.354 (3)
F13—C59	1.349 (3)	C34—F7C	1.354 (3)
F14—C59	1.342 (3)	C34—F7D	1.354 (3)
F15—C59	1.337 (3)	C34—F9B	1.372 (3)
F19—C50	1.337 (3)	C34—F9D	1.373 (3)
F20—C50	1.347 (3)	C34—F9C	1.373 (3)
F21—C50	1.329 (3)	C34—F9A	1.374 (3)
F22—C51	1.336 (3)	C36—C41	1.398 (3)
F23—C51	1.335 (3)	C36—C37	1.399 (3)
F24—C51	1.337 (3)	C36—B1	1.640 (3)
N1—C1	1.315 (3)	C37—C38	1.392 (3)
N1—C16	1.442 (3)	C38—C39	1.383 (3)
N1—C2	1.479 (3)	C38—C43	1.494 (3)
N2—C1	1.311 (3)	C39—C40	1.383 (3)
N2—C4	1.448 (3)	C40—C41	1.391 (3)
N2—C3	1.486 (3)	C40—C42	1.497 (3)
C2—C3	1.531 (3)	C44—C49	1.397 (3)
C4—C9	1.399 (3)	C44—C45	1.397 (3)
C4—C5	1.400 (3)	C44—B1	1.643 (3)
C5—C6	1.391 (3)	C45—C46	1.392 (3)
C5—C13	1.521 (3)	C46—C47	1.383 (3)
C6—C7	1.379 (3)	C46—C50	1.489 (3)
C7—C8	1.380 (3)	C47—C48	1.386 (3)
C8—C9	1.390 (3)	C48—C49	1.392 (3)
C9—C10	1.524 (3)	C48—C51	1.495 (3)
C10—C11	1.531 (3)	C52—C57	1.392 (3)
C10—C12	1.532 (3)	C52—C53	1.406 (3)
C13—C15	1.527 (3)	C52—B1	1.646 (3)
C13—C14	1.533 (3)	C53—C54	1.393 (3)
C16—C21	1.398 (3)	C54—C55	1.386 (3)
C16—C17	1.399 (3)	C54—C58	1.494 (3)
C17—C18	1.391 (3)	C55—C56	1.380 (3)
C17—C22	1.524 (3)	C56—C57	1.396 (3)
C18—C19	1.380 (3)	C56—C59	1.491 (3)
C19—C20	1.378 (3)	C58—F17A	1.343 (3)
C20—C21	1.397 (3)	C58—F17C	1.343 (3)
C21—C25	1.517 (3)	C58—F17B	1.343 (3)
C22—C23	1.522 (3)	C58—F17D	1.343 (3)
C22—C24	1.535 (3)	C58—F18D	1.355 (3)
C25—C27	1.529 (3)	C58—F18A	1.356 (3)
C25—C26	1.530 (4)	C58—F18C	1.356 (3)
C28—C29	1.400 (3)	C58—F18B	1.356 (3)
C28—C33	1.402 (3)	C58—F16B	1.363 (3)
C28—B1	1.638 (3)	C58—F16D	1.363 (3)
C29—C30	1.390 (3)	C58—F16C	1.364 (3)
C30—C31	1.385 (3)	C58—F16A	1.364 (3)
			
C1—N1—C16	127.50 (17)	C39—C38—C37	120.4 (2)
C1—N1—C2	108.78 (17)	C39—C38—C43	119.9 (2)
C16—N1—C2	123.64 (16)	C37—C38—C43	119.6 (2)
C1—N2—C4	127.33 (17)	C40—C39—C38	118.7 (2)
C1—N2—C3	108.32 (16)	C39—C40—C41	120.5 (2)
C4—N2—C3	121.63 (16)	C39—C40—C42	118.38 (19)
N2—C1—N1	114.83 (18)	C41—C40—C42	121.1 (2)
N2—C1—Cl1	122.96 (16)	C40—C41—C36	122.1 (2)
N1—C1—Cl1	122.21 (16)	F2—C42—F1	106.87 (19)
N1—C2—C3	102.43 (16)	F2—C42—F3	105.79 (19)
N2—C3—C2	102.55 (16)	F1—C42—F3	105.11 (18)
C9—C4—C5	123.8 (2)	F2—C42—C40	113.68 (18)
C9—C4—N2	118.65 (19)	F1—C42—C40	112.25 (19)
C5—C4—N2	117.44 (19)	F3—C42—C40	112.51 (18)
C6—C5—C4	116.6 (2)	F4—C43—F6	106.8 (2)
C6—C5—C13	121.7 (2)	F4—C43—F5	106.0 (2)
C4—C5—C13	121.67 (19)	F6—C43—F5	105.6 (2)
C7—C6—C5	121.1 (2)	F4—C43—C38	112.2 (2)
C6—C7—C8	120.8 (2)	F6—C43—C38	113.39 (19)
C7—C8—C9	121.0 (2)	F5—C43—C38	112.3 (2)
C8—C9—C4	116.8 (2)	C49—C44—C45	115.90 (19)
C8—C9—C10	119.3 (2)	C49—C44—B1	123.66 (17)
C4—C9—C10	123.96 (19)	C45—C44—B1	120.16 (18)
C9—C10—C11	111.30 (18)	C46—C45—C44	122.49 (19)
C9—C10—C12	111.1 (2)	C47—C46—C45	120.54 (19)
C11—C10—C12	110.6 (2)	C47—C46—C50	120.6 (2)
C5—C13—C15	113.4 (2)	C45—C46—C50	118.8 (2)
C5—C13—C14	110.42 (19)	C46—C47—C48	118.09 (19)
C15—C13—C14	110.0 (2)	C47—C48—C49	121.08 (19)
C21—C16—C17	123.61 (19)	C47—C48—C51	118.68 (19)
C21—C16—N1	118.76 (18)	C49—C48—C51	120.18 (18)
C17—C16—N1	117.60 (19)	C48—C49—C44	121.89 (19)
C18—C17—C16	116.9 (2)	F21—C50—F19	105.88 (19)
C18—C17—C22	120.4 (2)	F21—C50—F20	106.4 (2)
C16—C17—C22	122.61 (19)	F19—C50—F20	105.0 (2)
C19—C18—C17	121.1 (2)	F21—C50—C46	113.6 (2)
C20—C19—C18	120.6 (2)	F19—C50—C46	113.8 (2)
C19—C20—C21	121.2 (2)	F20—C50—C46	111.48 (19)
C20—C21—C16	116.6 (2)	F23—C51—F22	105.73 (17)
C20—C21—C25	120.4 (2)	F23—C51—F24	106.28 (18)
C16—C21—C25	123.01 (19)	F22—C51—F24	106.02 (18)
C23—C22—C17	113.0 (2)	F23—C51—C48	112.81 (18)
C23—C22—C24	110.9 (2)	F22—C51—C48	113.24 (17)
C17—C22—C24	110.26 (19)	F24—C51—C48	112.18 (17)
C21—C25—C27	110.75 (19)	C57—C52—C53	115.63 (19)
C21—C25—C26	111.4 (2)	C57—C52—B1	123.57 (18)
C27—C25—C26	111.1 (2)	C53—C52—B1	120.50 (18)
C29—C28—C33	115.55 (19)	C54—C53—C52	122.39 (19)
C29—C28—B1	122.78 (18)	C55—C54—C53	120.5 (2)
C33—C28—B1	121.23 (18)	C55—C54—C58	119.43 (19)
C30—C29—C28	122.4 (2)	C53—C54—C58	120.02 (19)
C31—C30—C29	120.7 (2)	C56—C55—C54	118.17 (19)
C31—C30—C34	120.2 (2)	C55—C56—C57	121.10 (19)
C29—C30—C34	119.0 (2)	C55—C56—C59	120.39 (19)
C30—C31—C32	118.2 (2)	C57—C56—C59	118.50 (19)
C33—C32—C31	120.6 (2)	C52—C57—C56	122.2 (2)
C33—C32—C35	120.2 (2)	F15—C59—F14	106.54 (18)
C31—C32—C35	119.1 (2)	F15—C59—F13	105.96 (18)
C32—C33—C28	122.5 (2)	F14—C59—F13	104.88 (18)
F8B—C34—F7B	103.9 (5)	F15—C59—C56	113.22 (19)
F8A—C34—F7A	109.7 (4)	F14—C59—C56	112.99 (18)
F8C—C34—F7C	102.1 (7)	F13—C59—C56	112.58 (19)
F8D—C34—F7D	95.3 (12)	C28—B1—C36	111.67 (17)
F8B—C34—F9B	113.8 (5)	C28—B1—C44	103.48 (16)
F7B—C34—F9B	108.1 (3)	C36—B1—C44	111.86 (16)
F8D—C34—F9D	115.6 (13)	C28—B1—C52	113.14 (16)
F7D—C34—F9D	96.9 (12)	C36—B1—C52	104.19 (16)
F8C—C34—F9C	105.0 (5)	C44—B1—C52	112.77 (17)
F7C—C34—F9C	105.2 (8)	F17D—C58—F18D	100.2 (9)
F8A—C34—F9A	98.6 (5)	F17A—C58—F18A	106.0 (3)
F7A—C34—F9A	101.7 (4)	F17C—C58—F18C	103.6 (8)
F11—C35—F12	106.67 (19)	F17B—C58—F18B	107.2 (4)
F11—C35—F10	105.42 (18)	F17B—C58—F16B	109.1 (3)
F12—C35—F10	106.10 (19)	F18B—C58—F16B	105.7 (4)
F11—C35—C32	112.32 (19)	F17D—C58—F16D	106.2 (9)
F12—C35—C32	112.96 (19)	F18D—C58—F16D	111.7 (9)
F10—C35—C32	112.80 (18)	F17C—C58—F16C	109.5 (8)
C41—C36—C37	116.00 (19)	F18C—C58—F16C	98.7 (8)
C41—C36—B1	123.41 (18)	F17A—C58—F16A	106.8 (3)
C37—C36—B1	120.42 (18)	F18A—C58—F16A	104.3 (3)
C38—C37—C36	122.2 (2)		

### Hydrogen-bond geometry (Å, º)

**Table:**

*D*—H···*A*	*D*—H	H···*A*	*D*···*A*	*D*—H···*A*
C3—H3*A*···F15^i^	0.99	2.43	3.256 (3)	141
C12—H12*A*···F17*C*^ii^	0.98	2.53	3.269 (12)	132
C19—H19···F8*D*^iii^	0.95	2.48	3.297 (18)	144
C29—H29···F9*D*^iii^	0.95	2.35	3.180 (14)	146

Symmetry codes: (i) −*x*+1/2, *y*−1/2, −*z*+1/2; (ii) *x*−1/2, −*y*+1/2, *z*+1/2; (iii) −*x*+1, −*y*+1, −*z*+1.
